# Targeting the Endothelin-1 pathway to reduce invasion and chemoresistance in gallbladder cancer cells

**DOI:** 10.1186/s12935-023-03145-9

**Published:** 2023-12-10

**Authors:** Francisco Rodas, Jetzabel A. Vidal-Vidal, Daniela Herrera, David A. Brown-Brown, Diego Vera, Joaquín Veliz, Pilar Püschel, José I. Erices, Verónica Sánchez Hinojosa, Julio C. Tapia, Eduardo Silva-Pavez, Claudia Quezada-Monrás, Pablo Mendoza-Soto, Flavio Salazar-Onfray, Cristian Carrasco, Ignacio Niechi

**Affiliations:** 1 Instituto de Bioquímica y Microbiología, Facultad de Ciencias, Universidad Austral de Chile, Valdivia, Chile; 2 Millennium Institute on Immunology and Immunotherapy, Instituto de Bioquímica y Microbiología, Facultad de Ciencias, Universidad Austral de Chile, Valdivia, Chile; 3 Laboratorio de transformación celular, Programa de Biología Celular y Molecular, Instituto de Ciencias Biomédicas, Facultad de Medicina, Universidad de Chile, 8380453 Santiago, Chile; 4 Facultad de Odontología y Ciencias de la Rehabilitación, Universidad San Sebastián, Bellavista, Santiago, Chile; 5 Millennium Institute on Immunology and Immunotherapy, Faculty of Medicine, Universidad de Chile, 8380453 Santiago, Chile; 6 Disciplinary Program of Immunology, Institute of Biomedical Sciences, Faculty of Medicine, Universidad de Chile, 8380453 Santiago, Chile; 7 Subdepartamento de Anatomía Patológica, Hospital Base de Valdivia, 5090000 Valdivia, Chile

**Keywords:** Gallbladder cancer, Endothelin-1, Cell invasion, Chemosensitivity, Therapeutic targeting

## Abstract

**Background:**

Gallbladder cancer (GBC) is a prevalent and deadly biliary tract carcinoma, often diagnosed at advanced stages with limited treatment options. The 5-year survival rate varies widely from 4 to 60%, mainly due to differences in disease stage detection. With only a small fraction of patients having resectable tumors and a high incidence of metastasis, advanced GBC stages are characterized by significant chemoresistance. Identification of new therapeutic targets is crucial, and recent studies have shown that the Endothelin-1 (ET-1) signaling pathway, involving ET_A_R and/or ET_B_R receptors (ETRs), plays a crucial role in promoting tumor aggressiveness in various cancer models. Blocking one or both receptors has been reported to reduce invasiveness and chemoresistance in cancers like ovarian, prostate, and colon. Furthermore, transcriptomic studies have associated ET-1 levels with late stages of GBC; however, it remains unclear whether its signaling or its inhibition has implications for its aggressiveness. Although the role of ET-1 signaling in gallbladder physiology is minimally understood, its significance in other tumor models leads us to hypothesize its involvement in GBC malignancy.

**Results:**

In this study, we investigated the expression of ET-1 pathway proteins in three GBC cell lines and a primary GBC culture. Our findings demonstrated that both ET_A_R and ET_B_R receptors are expressed in GBC cells and tumor samples. Moreover, we successfully down-regulated ET-1 signaling using a non-selective ETR antagonist, Macitentan, which resulted in reduced migratory and invasive capacities of GBC cells. Additionally, Macitentan treatment chemosensitized the cells to Gemcitabine, a commonly used therapy for GBC.

**Conclusion:**

For the first time, we reveal the role of the ET-1 pathway in GBC cells, providing insight into the potential therapeutic targeting of its receptors to mitigate invasion and chemoresistance in this cancer with limited treatment options. These findings pave the way for further exploration of Macitentan or other ETR antagonists as potential therapeutic strategies for GBC management. In summary, our study represents a groundbreaking contribution to the field by providing the first evidence of the ET 1 pathway's pivotal role in modulating the behavior and aggressiveness of GBC cells, shedding new light on potential therapeutic targets.

**Supplementary Information:**

The online version contains supplementary material available at 10.1186/s12935-023-03145-9.

## Background

Gallbladder cancer (GBC) stands out as the most common and lethal form of biliary tract carcinoma [1]. Its 5-year survival rate varies widely, ranging from 4 to 60%. This significant range is primarily contingent on the stage at which the disease is detected, and unfortunately, in advanced stages, when patients typically receive a diagnosis, the 5-year life expectancy drops to less than 5% [1–3]. Incidence rates of GBC vary widely, with the highest cases reported annually in certain Eastern European countries, Asia, and Latin America [3]. The development of GBC is influenced by several risk factors, including gender, genetic-related geographic factors, chronic inflammation (cholecystitis), and gallstones (cholelithiasis) [4]. The elevated incidence rates in certain regions may be attributed to the high prevalence of cholelithiasis, especially in women, and the presence of genetic variants associated with the Mapuche ethnic group in South America, but the association between these risk factors has not been fully described and is still a subject of investigation [5]. However, it is thought that the common factor is related to chronic inflammation, though the exact origin and development of the pathology are not entirely clear.

The prognosis for GBC patients is grim, with an average survival time of 4 to 14 months, and the most effective treatment option being surgical resection [6]. However, less than 10% of patients have resectable tumors at the time of diagnosis, and nearly 50% already exhibit metastasis, frequently to the liver [7]. Even with surgical intervention, most patients progress to a metastatic stage [8], where the cancer cells exhibit significant resistance to conventional chemotherapy, with gemcitabine being the gold standard [9, 10]. Additionally, poorly differentiated GBC tissues, indicative of an invasive phenotype, are strongly associated with an increased risk of metastasis and poor patient outcomes [11, 12]. In GBC, the lack of effective treatment options and the challenges associated with late-stage diagnosis highlight the urgent need for novel therapeutic approaches. GBC's grim prognosis underscores the critical requirement for innovative treatments to improve patient outcomes.

The malignant progression of GBC, characterized by dedifferentiation traits, is regulated by several signaling pathways that promote epithelial to mesenchymal transition (EMT), cell migration, invasiveness, and metastasis [13, 14]. These pathways include β-catenin, Hedgehog, TGF-β, PI3K/AKT, mTOR, among others plausible molecular targets [15–19]. Within the microenvironment of solid tumors, such as GBC, various autocrine and paracrine signaling molecules enhance tumor malignancy, among them Tumor Necrosis Factor-alpha (TNFα), Vascular Endothelial Growth Factor (VEGF), and Endothelin-1 (ET-1) [20–22]. While ET-1 is a well-known peptide involved in vasoconstriction and gallbladder physiology [23, 24], it has also been linked to cell survival, proliferation, angiogenesis, invasion, and metastasis in several cancers [22–28].

ET-1 signaling is mediated by two G protein-coupled receptors, ET_A_R and ET_B_R, which activate downstream pathways such as PLCβ, leading to calcium mobilization, PKC activation, and nuclear import of β-catenin and NF-κB [29–32]. ET-1 signaling target genes related to cancer progression include CCND1 (Cyclin-D1), AXIN2, PTGS2 (COX2), VEGF, ZEB1, and EDN1 (ET-1) itself [31, 33]. Elevated levels of ET-1 have been observed in certain cancers with invasive phenotypes, correlating with reduced survival and indicating its potential as a prognostic marker [34–36]. Genomic and transcriptome studies have revealed the expression of ET-1 and ETRs in biliary tract carcinomas, including GBC, with their levels correlating with advanced tumor stages [37, 38]. Macitentan, a non-selective dual ET_A_R/ET_B_R antagonist, FDA-approved for pulmonary hypertension, has shown promise in preclinical studies for various cancers [39–43]. While ET-1's role in various cancers has been extensively studied, its potential significance in GBC is a subject of growing interest. ET-1 has been demonstrated to play a crucial role in cancer progression in several malignancies, including prostate [44], colon [45], ovarian [31], lung [31], pancreatic [46], and others. Studies have shown that ET-1 can promote invasion and metastasis in these cancer types, suggesting that it may exert similar effects in GBC.

Considering the limited treatment options and poor prognosis associated with GBC [47], the role of ET-1 signaling emerges as a promising avenue for further investigation. Its significance as a prognostic marker and therapeutic target in other cancer types adds to its potential importance [48]. Thus, the main objective of this study is to explore the presence and functional role of the ET-1 signaling pathway in GBC in vitro. Through our investigation, we aim to provide valuable insights that may contribute to the development of improved treatment strategies for this challenging malignancy.

## Materials and methods

### Tumor samples and immunohistochemistry

A retrospective analysis was conducted on cholecystectomy specimens diagnosed with GBC, along with corresponding clinical data from patients at the Pathological Anatomy Subdepartment of Hospital Base Valdivia, Chile, spanning 2001 to 2018. The study, encompassing 180 cases, exclusively focused on primary invasive gallbladder adenocarcinoma, excluding in situ adenocarcinoma, squamous carcinoma, neuroendocrine carcinoma, and metastases. Ethical approval was obtained from the Valdivia Bioethical Committee for Human Research. Tissue Microarrays (TMAs), previously constructed by our laboratory [12], were utilized. These TMAs, containing positive tissue controls, were subjected to immunohistochemical analysis using an automatic BenchMark GX Ventana system (Roche, AZ, USA). Primary antibodies were purchased from Abcam, ET_A_R (ab219358, 1:200) and ET_B_R (ab230618 1:2000). The ultraView Universal DAB Detection kit (Roche, Arizona, USA) was employed as per the manufacturer's instructions. Two independent pathologists evaluated immunohistochemistry slides blindly under light microscopy, categorizing antigen expression based on positive and tissue controls within each slide. Antigen expression intensity was graded subjectively, considering positive tissue controls, and categorized as negative expression. Kaplan–Meier curves were constructed based on the presence or absence of positive staining.

### Cell culture and treatments

Three GBC cell lines (NOZ, TGBC-1TKB, TGBC-2TKB) and one primary culture (CAVE1) were used in this study. NOZ line is derived from ascites metastasis [49]. 1TKB line was derived from a lymph node of a gallbladder adenocarcinoma, and 2TKB was derived from a primary lesion of the same patient as 1TKB [50]. CAVE1 was obtained from a primary GBC tumor from a Chilean patient [51]. Once arrived at laboratory, all cells were immediately expanded in DMEM-HG medium supplemented with 10% FBS, 100 U/ml penicillin and 100 μg/ml streptomycin (Gibco) at 37 °C and 5% CO_2_, followed by storage in liquid nitrogen at − 190 °C. Once a year, one nitrogen aliquot was thawed, expanded, and stored again at − 80 °C. For experiments, one − 80 °C aliquot was thawed and grown in standard conditions. All experiments were performed within 1 year and cells were eliminated after 15 passages, as requested by each local biosecurity committee. Mycoplasma contamination was tested monthly with the EZ-PCR Mycoplasma Test kit (Biological Industries, Beit Haemek, Israel), being the last test performed 6 months ago and yielding no contamination. Macitentan (MedChemExpress) was used at 1 µM and ET-1 (Sigma Aldrich) at 100 nM.

### Enzyme-linked immunosorbent assay (ELISA)

ET-1 secreted to culture medium was quantified using Endothelin-1 (ET-1) Human ELISA Kit (Thermofisher, EIAET1). A cell density of 10^4^ cells/well were incubated for 48 h in serum free DMEM-HG. ET-1 levels (pg) were measured according to the manufacturer’s instructions and normalized to total protein content and cell number.

### 3D-migration and invasion

Cells (5 × 10^4^ cells/chamber) were plated on the upper side of a polycarbonate Transwell chamber (6.5 mm, 8.0 μm, Corning, Lowell, MA, USA) for migration assay or in a 300 µg/ml matrigel-coated Transwell chamber for invasion assay with 2 µg/ml fibronectin in the bottom side to promote cell attachment. In both cases, cells were seeded in serum-free DMEM-HG. As chemoattractant, the bottom chamber contained DMEM-HG supplemented with 10% FBS. Cells were incubated at 37 °C for 4 h (migration) or 16 h (invasion). Cells in the top chamber were carefully removed with cotton swabs and cells that crossed through the chamber were fixed with 0.5% crystal violet solution in 10% Methanol for 10 min at room temperature. Cells were counted using the 10× objective in 5 different fields of the underside of the insert. The mean number of cells was normalized to 1 using the control condition and then plotted.

### RT-qPCR

Total RNA was extracted with TRIzol (Gibco) and quantified by NanoDrop. Reverse transcription was performed with 1 µg RNA plus M-MLV RT (Promega) following manufacturer instructions. qPCR was performed in a Stratagene MX30005P (Agilent Technologies Inc), using the ΔΔCt method and ACTB (β-actin) as a normalizer gene. For the reaction, buffer 2 × Master mix qPCR Brilliant II Sybr® Green (ThermoFisher, Waltham, MA, USA) was used, following the manufacturer’s instructions. Primers sequences are listed in Additional file 1: Table S1.

### Western blot

Proteins (30–40 μg) were separated by SDS-PAGE (BioRad, Hercules, CA, USA), transferred to a 0.22 μm nitrocellulose membrane and then blocked with 5% non-fat milk in PBS-Tween 0.05%. Membranes were incubated at 4 °C overnight with primary antibodies followed by incubation for 1 h with a secondary HRP-conjugated anti-IgG antibody (Jackson Laboratories, 1:50,000 in 1X PBS-Tween 0.05%). Primary antibodies were Snail (CST #3879, 1:1000), E-cadherin (CST #3195, 1:1000), β-actin (Santa Cruz Biotechnology #47778, 1:5000), MMP9 (CST #13667, 1:1000), ZEB1 (CST #3396, 1:1000), Lamin B1 (CST #12586, 1:1000), β-catenin (BD Biosciences #610153, 1:1000), NF-kB (CST #6956, 1:1000), ETAR (Thermo Scientific™ #PA3-065, 1:1000), ETBR (Thermo Scientific™ #PA3-066, 1:1000). Bands were revealed using the West Dura chemiluminescence system (Thermo-Fisher) and imaging was performed on a Syngene G:Box instrument (Synoptics, Cambridge, UK).

### Reporter assay

NOZ cells were transfected with 10 µg of total DNA of pTOP-FLASH or pFOP-FLASH. Cells were lysed 24 h post-transfection with pasive lysis buffer. Luciferase activity was measured with luciferin substrate (Promega), following instructions provided by the manufacturer. The values reported for luciferase activity for each condition were used for calculating the TOP-FLASH/FOP-FLASH activity ratios. Values shown were averaged from at least three independent experiments.

### Protein stability

NOZ cells (10^6^) were cultured in standard conditions and incubated with 20 µg/ml cycloheximide (CHX) in the absence or presence of 1 µM MAC and/or 100 nM ET-1. Cells were harvested after 0, 0.5, 1, 2 and 4 h of treatment. Cell extracts were analyzed by Western blot, using an anti-β-catenin antibody.

### Indirect immunofluorescence (IFI)

Cells (2.5 × 10^4^) were grown on glass coverslips and treated with 1 µM MAC and/or 100 nM ET1 for 24 h. Samples were and fixed with PBS/4% paraformaldehyde and incubated with anti-β-catenin specific antibody (BD #610153) and DAPI for nuclear staining. Alexa fluor 594 anti-mouse (Thermofisher) was used as a secondary antibody. Coverslips were mounted onto slides with DAKO and fluorescence was visualized with a Zeiss AxioObserver microscope.

### Cell viability

CellTiter 96AQueous One Solution Cell Proliferation Assay (MTS) from Promega (Madison, WI, USA) was performed following manufacturer instructions. Briefly, 2.5 × 10^4^ cells were seeded in 96-well plates for 24 h and treated with Gemcitabine (0–100 μM) alone or in combination with 1 μM Macitentan (MAC) for 72 h. Cells were incubated with MTS reagent for 2 h and absorbance was measured at 490 nm using a microplate reader (Synergy HT, BioTek Instruments, Inc.). Alternatively, for crystal violet viability assays, 5 × 10^3^ cells (NOZ and 2TKB) were seeded and treated with MAC/GEM for 72 h in the presence or absence of 1 mM pyruvate. Viability was indirectly measured by violet crystal stain and quantified by absorbance at 570 nm and plotted as percentage.

### Statistical analysis

Statistical analysis and graphical representations were conducted using GraphPad Prism 8.1 software. Values were presented as mean ± SD from a minimum of three independent experiments. Statistical analysis was performed on normalized data using the unpaired t-Student test for unpaired data and one-way ANOVA for data groups. Kaplan–Meier and log-rank tests (Mantel-Cox) were employed to construct and assess survival data. P ≤ 0.05 was considered statistically significant."

## Results

### ET-1 axis characterization in gallbladder cancer cells

The function of ET-1 in gallbladder physiology has been already partially described, however, the aberrant secretion is related to gallbladder inflammation, as well as its transcript is increased in GBC samples, but its role in GBC progression is unknown [37, 38].

**Fig. 1:**
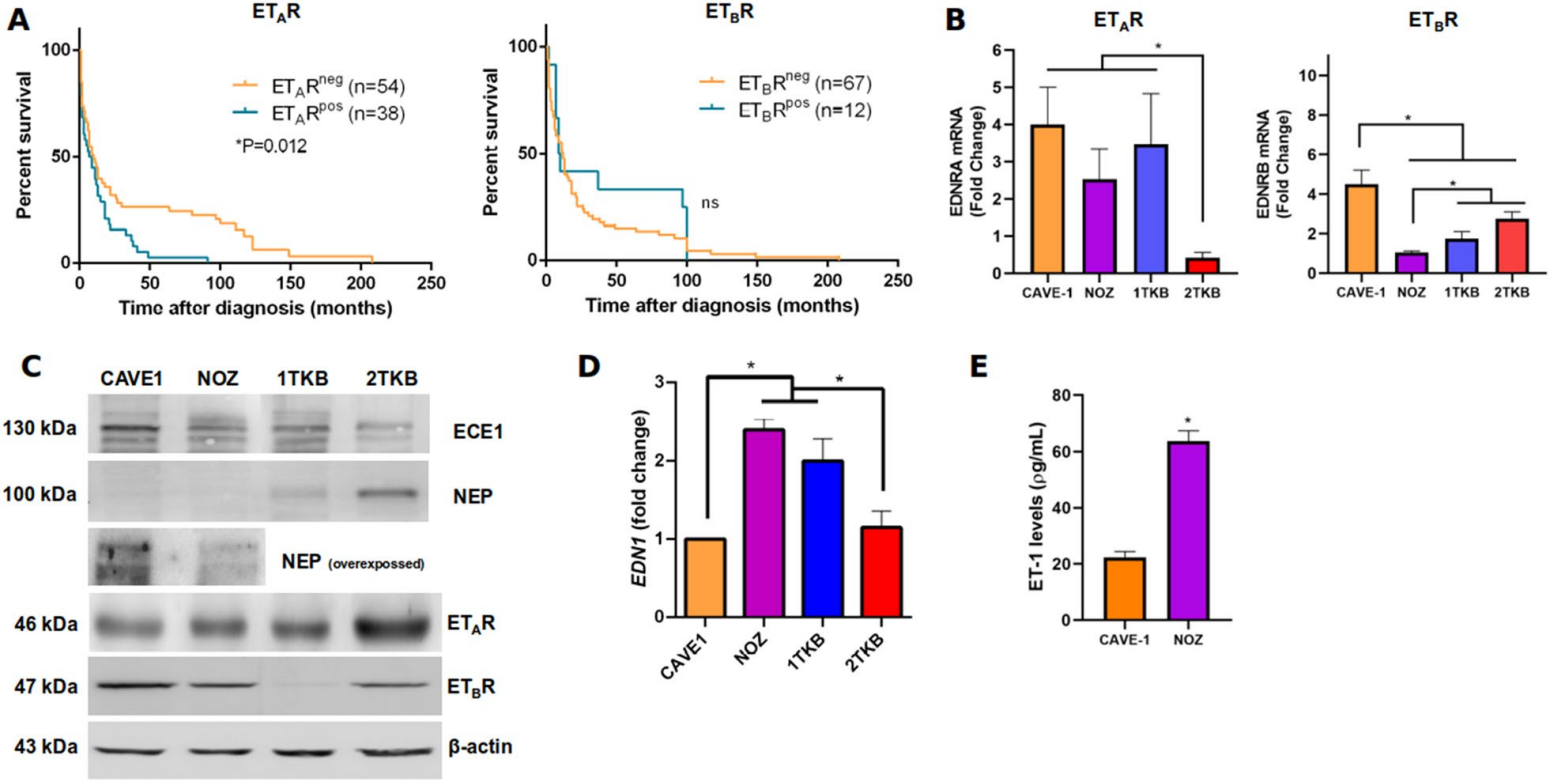
ET-1 and its receptors are expressed in GBC cells. **a** Kaplan–Meier post-diagnosis overall-survival (OS) estimation of GBC patients, according to ET-1 receptors expression. Each graph shows the number of patients in each group (n), the median OS time in months, and overall survival rate (%). p-values were calculated using a log-rank (Mantel–Cox) test. **b** ET-1 receptors, ET_A_R (*EDNRA*) and ET_B_R (*EDNRB*), transcripts levels were measured by RT-qPCR in four GBC cell lines. **c** ET-1 receptors ET_A_R and ET_B_R, ECE1 and NEP protein levels were detected by western blot in four GBC cell lines. Representative images from three independent experiments are shown. **d** ET-1 transcripts (*EDN1*) levels were measured by RT-qPCR in four GBC cell lines. **e** ET-1 extracellular levels (pg/ml) were measured by ELISA in CAVE-1 and NOZ GBC cells. Data represent averages ± SEMs (n = 3). ANOVA and Student’s tests were used. *p < 0.05

We analyzed the association between ET-1 receptors expression and median overall survival of GBC patients using TMAs. We found a significant relationship between ET_A_R positive samples with less survival, suggesting this receptor as a possible marker of poor prognosis. No relationship between ET_B_R positive samples with overal survival were founded (Fig. 1a). In order to characterize the ET-1 signaling pathway in our cells line, ET_A_R and ET_B_R levels were measured by RT-qPCR (Fig. 1b) and western blot (Fig. 1c) in 4 GBC cells, showing that both receptors are expressed in all cells lines. Notably, ET_B_R is less expressed in 1TKB cells of a lymph node metastatic origin, suggesting a predominant role of ET_A_R in GBC progression. However, both receptor’s levels are well detectable in the other cell lines, including primary cultures (CAVE1). ET-1 processing enzymes levels were also detected in four cell lines highlighting that the enzyme which degrades ET-1 (NEP) is overexpressed in non-metastasic 2TKB cells, while the enzyme that activates ET-1 (ECE1) is diminished (Fig. 1c). ET-1 mRNA (EDN1) levels were detected in four cell lines, showing higher levels in NOZ and 1TKB cells, which share a metastasic origin (Fig. 1d). Finally, ET-1 levels were measured by ELISA in two cell lines showing that ET-1 levels are three-fold higher in NOZ cells in comparison with CAVE1 derived from a primary gallbladder adenocarcinoma, suggesting a role of ET-1 in GBC progression (Fig. 1e). These data show for the first time the expression of some ET-1 signaling members, suggesting a function related to the malignant progression of this cancer.

### Macitentan downregulates ET-1 signaling pathway in gallbladder cancer

**Fig. 2:**
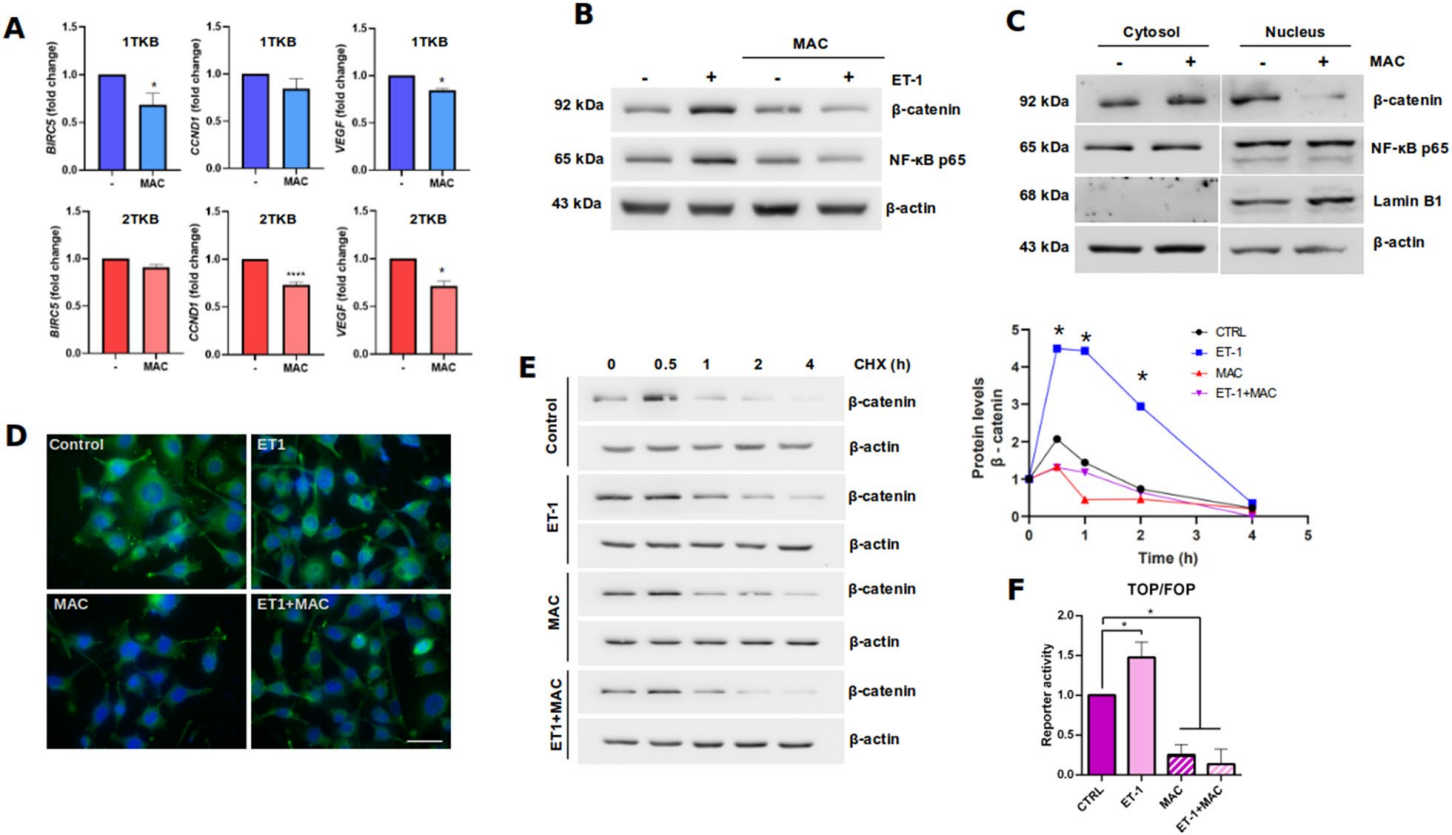
Dual ETRs antagonism with macitentan (MAC) promotes ET-1 signaling downregulation in GBC cells. **a** Transcript levels of ET-1 target genes were measured by RT-qPCR in two GBC cell lines, 1TKB (blue) and 2TKB (red) treated with 1 µM MAC for 24 h. **b** Total β-catenin and NF-kB (p65) levels were measured by western blot in NOZ GBC cells treated with 100 nM ET-1 and/or 1 µM MAC for 24 h. Representative images from three independent experiments are shown. **c** Cytosolic and nuclear β-catenin and NF-kB (p65) were measured by western blot in 1TKB and 2TKB GBC cells treated with 1 µM MAC for 24 h. Lamin B1 was used as a nuclear marker. **d** Indirect immunofluorescence of NOZ cells treated with 1 µM MAC and/or 100 nM ET1 for 24 h. β-catenin was visualized using a specific antibody and the nucleus was stained with DAPI. **e** Cycloheximide stability assay of β-catenin in NOZ cells treated with 100 nM ET-1 and/or 1 µM MAC between 0 and 4 h. **f** Reporter activity of β-catenin was measured by luciferase reporter assay using the TOP/FOB Flash system. Values reported for luciferase activity were used to calculate the TOP/FOP ratios (mean ± SD). Representative images from three independent experiments are shown. Data represent averages ± SDs (n = 3). ANOVA and Student’s tests were used. *p < 0.05

To evaluate the sensitivity of GBC cells to ET-1/ETRs blockade, the cells were treated with the dual antagonist macitentan (MAC) and different features that account for ET-1 downregulation were evaluated. Transcript levels of ET-1 target genes were analyzed in two cell lines derived from the same patient but with different origins, 1TKB (lymph node metastasis) and 2TKB (primary tumor). Results showed that several target genes involved in GBC progression were downregulated by MAC, with the greatest effect on 1TKB cells which have a more aggressive origin (Fig. 2a). NF-kB and β-catenin protein levels were evaluated as markers of ET-1/ETRs activation. Protein levels of these proteins were induced by ET-1, and this was blocked by MAC (Fig. 2b). Also, nuclear β-catenin and NF-kB decreased under ETRs blockage in 1TKB and 2TKB cells, respectively (Fig. 2c, d). Interestingly, β-catenin levels were augmented in the cytosolic fraction of 2TKB cells (Fig. 2c). Since it is known that protein levels of β-catenin are mainly regulated its degradation, a Cycloheximide assay was performed to assess the stability of the protein. ET-1 was shown to increase the stability of β-catenin, which is prevented with MAC (Fig. 2e). ET-1 is capable of increasing the reporter activity of β-catenin, which is reversed when its receptors are blocked with MAC (Fig. 2f). All these results suggest that the ET-1 pathway is active in GBC cells, but importantly it can be downregulated by the antagonist MAC.

### ET-1/ETRs blockade decreases migration and invasion

**Fig. 3:**
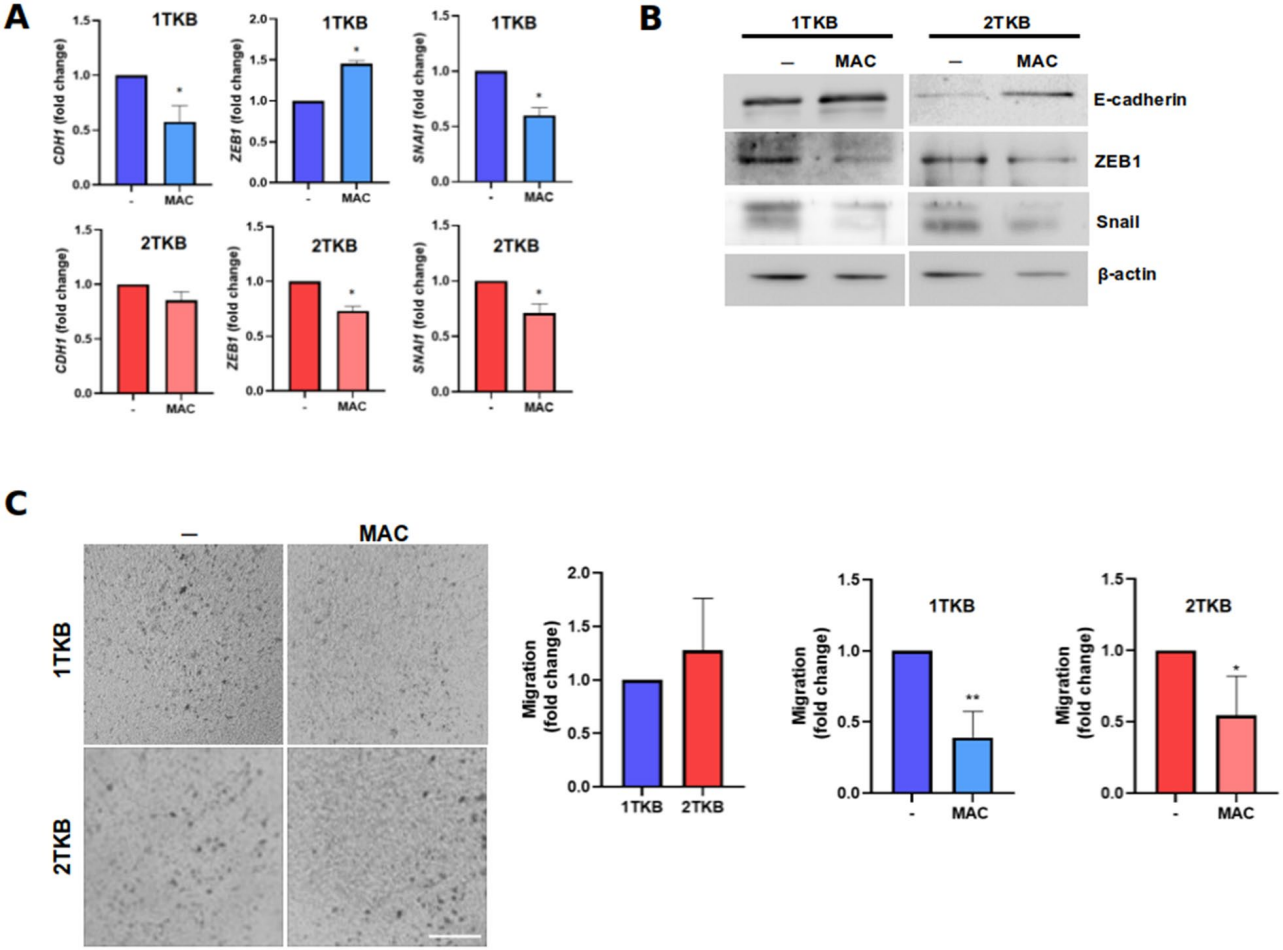
Blockade of ET-1 signaling decreases EMT-marker expression and 3D-cell migration. **a** Transcript levels of EMT markers were measured by RT-qPCR in two GBC cell lines, 1TKB (blue) and 2TKB (red), treated with 1 µM MAC for 24 h. **b** Protein levels of EMT markers were measured by western blot in two GBC cell lines, 1TKB and 2TKB, treated with 1 µM MAC for 24 h. **c** 1TKB and 2TKB cells were seeded in a transwell chamber in FBS free medium with 10% FBS in the lower bottom as a chemoattractant. Cells were incubated at 37 °C for 4 h in presence or absence of 1 µM MAC. Cells were fixed and stained with crystal violet and counted using the 10× objective in 5 different fields. Control was normalized to 1 and finally plotted as fold changes. Scale bar: 100 nm. Data represent averages ± SDs (n = 3). ANOVA and Student’s tests were used. *p < 0.05 **p < 0.01

**Fig. 4:**
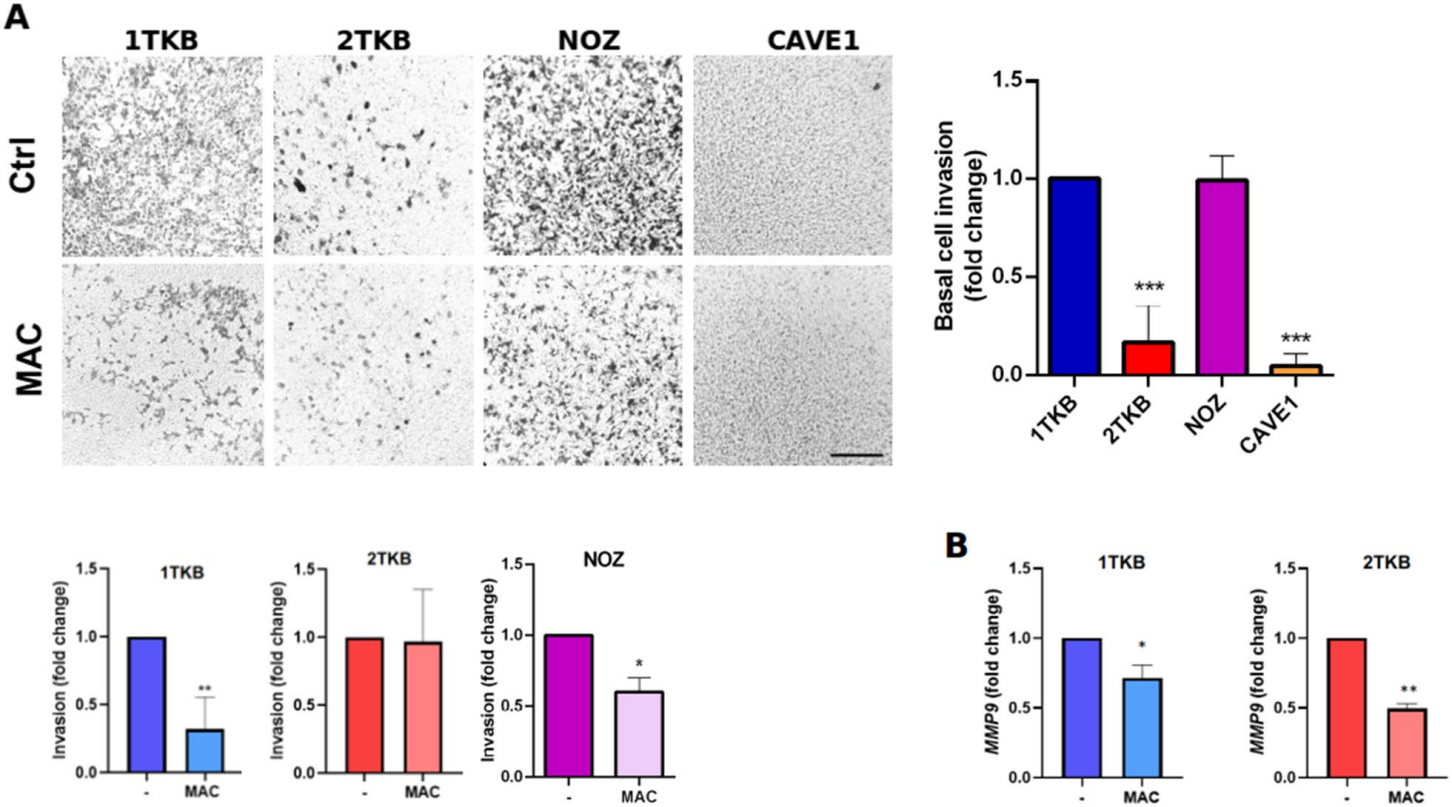
ET-1 signaling blockage with macitentan decreases in vitro GBC cell invasion. **a** 1TKB (blue), 2TKB (red), NOZ (purple) and CAVE1 (yellow) cells were seeded in a matrigel-coated transwell chamber in FBS free medium, while the lower bottom contained medium + 10% FBS as chemoattractant. Cells were incubated at 37 °C for 16 h in presence or absence of 1 µM MAC. Cells were fixed and stained with crystal violet and counted using the 10× objective in 5 different fields. Control was normalized to 1 and then plotted as fold changes. Scale bar: 100 nm. Data represent averages ± SEMs (n = 3). ANOVA and Student’s tests were used. *p < 0.05 **p < 0.01. **b** MMP9 transcript levels were measured by RT-qPCR in two GBC cell lines, 1TKB (blue) and 2TKB (red), treated with 1 µM MAC for 24 h. Data represent averages ± SDs (n = 3)

It has been described that ET-1 is a mitogenic peptide capable of promoting EMT, cell migration and invasion [29–33], however, it has not been studied in GBC. Therefore, we evaluated the effect of ET-1/ETRs blockade on the expression of EMT markers, as well as the migratory capacity of GBC cells. Results show that Slug, Snail and ZEB1 levels decreased in response to MAC, while E-cadherin increased, as expected (Fig. 3a, b). In order to evaluate the basal migratory capacity, cells were seeded in transwell chambers for 6 h, showing that both cells showed a similar capacity to cross the porous membrane (Fig. 3c). Notably, MAC treatment was able to decrease the migration in all lines tested, suggesting that ET-1 pathway regulates GBC cell migration, which is a critical step in metastatic progression. To exclude any potential effects of proliferation in migration capacity results, we evaluated the effect of MAC on proliferation, showing that even at longer times, blockade of ETRs does not affect viability, either in the presence or absence of pyruvate (Additional file 1: Fig. S1). Considering the observed effects of MAC on migration, we evaluated its effect on ET-1-mediated invasion. To evaluate the basal invasive capacity, cells were seeded in transwell/matrigel chambers for 16 h. The results showed that 1TKB and NOZ cells have a higher invasive capacity (Fig. 4a), which agree with its aggressive origin compared to 2TKB and CAVE1. Additionally, treatment with MAC decreased the invasiveness of 1TKB and NOZ, but not 2TKB (Fig. 4a). Regarding CAVE1, there are no discernible variances in treatment, as the basal invasion is exceedingly minimal, to the point of being nearly imperceptible. This outcome aligns seamlessly with its inherently non-invasive nature. Despite these results, MMP9 transcripts levels were similarly decreased by MAC in two cell lines (Fig. 4b).

### Macitentan sensitizes gallbladder cancer cells to gemcitabine

**Fig. 5:**
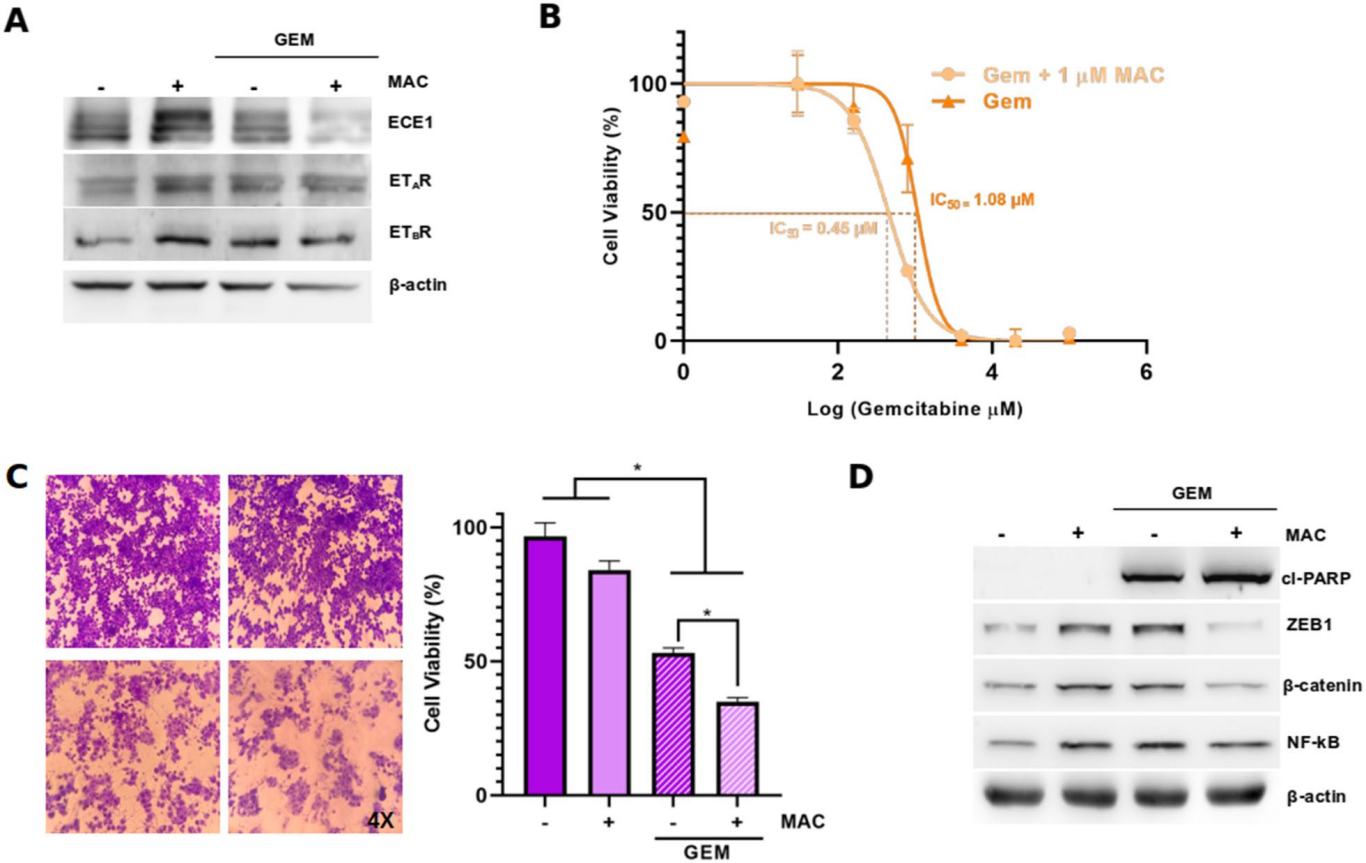
ET-1 signaling blockage with macitentan sensitizes GBC cells to gemcitabine. **a** ECE1, ET_A_R and ET_B_R protein levels were detected by western blot in NOZ cells treated with 14 µM GEM and/or 1 µM MAC for 24 h. **b** Cells were seeded in 96-well plates for 24 h and treated with increased concentrations of gemcitabine (GEM) alone or in combination with 1 μM MAC for 72 h. Cells were incubated with MTS reagent for 2 h and absorbance was measured at 490 nm and plotted as percentage. **c** Cell viability of NOZ cells treated with 14 µM GEM and/or 1 µM MAC for 72 h measured by crystal violet assay. **d** ZEB1, β-catenin, NF-kB and cleaved PARP (cl-PARP) detection by western blot in NOZ cells treated with 14 µM GEM and/or 1 µM MAC for 24 h. Data represent averages ± SDs (n = 3). ANOVA test was used. *p < 0.05

**Fig. 6:**
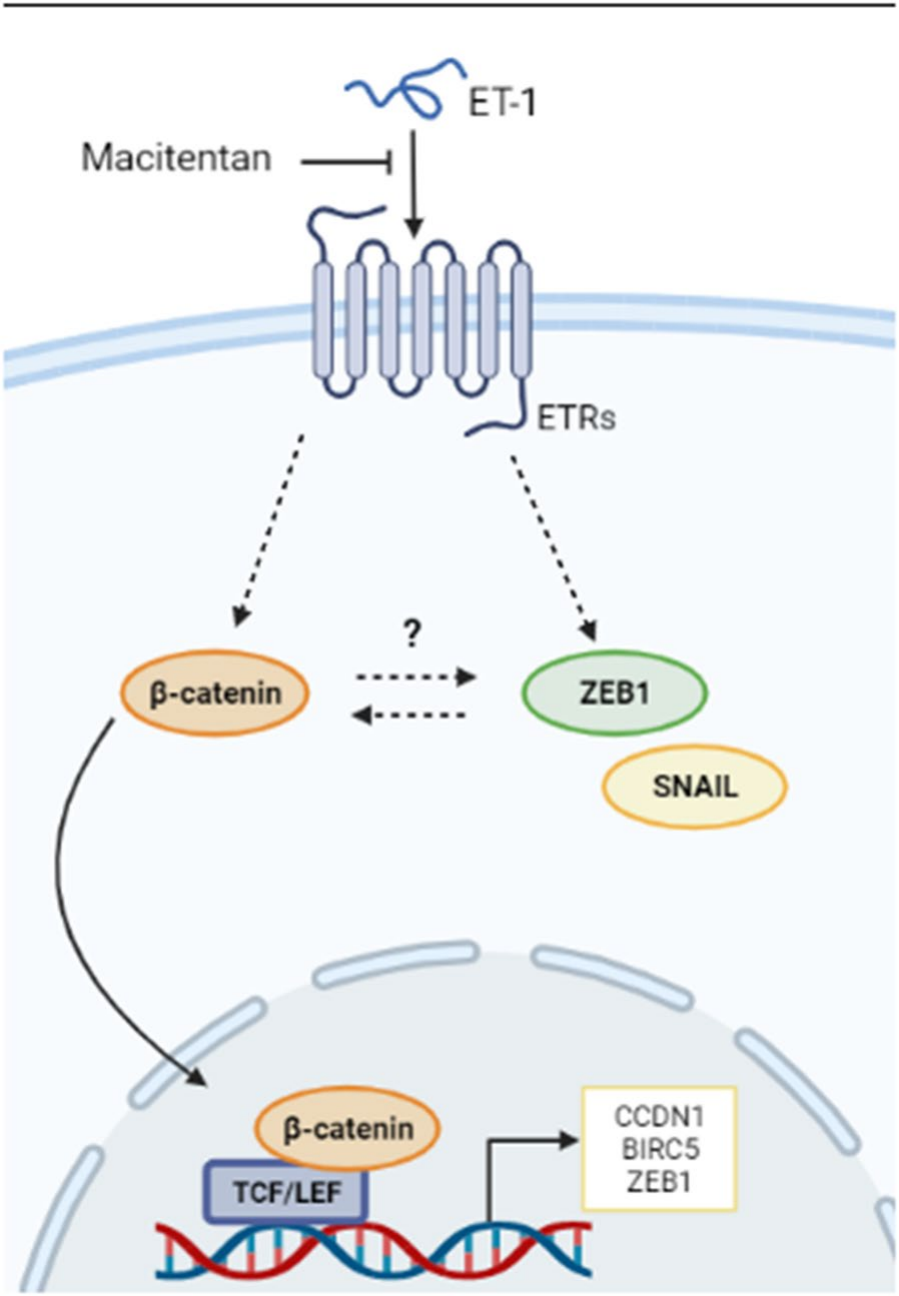
Potential use of blocking ET-1 signaling with macitentan in gallbladder cancer therapy. ETRs blockage with macitentan regulates ET-1 target genes expression and nuclear protein levels of β-catenin. ET-1 signaling blockage downregulates EMT markers (Snail and ZEB1), impairing cell invasion

GBC cells are highly resistant to conventional therapies, such as gemcitabine (GEM) [9, 10]. Thus, we evaluated by MTS whether ETRs blockade with MAC could sensitize GBC primary cells (CAVE1) and a highly chemoresistant cell line (NOZ) to GEM. Importantly, MAC itself was not cytotoxic for at least 72 h (Additional file 1: Fig. S1). Our results showed that treatment with GEM induce both, ET_A_R and ET_B_R levels, suggesting a possible role of ET-1 signaling in GEM effectivity (Fig. 5a). Furthermore, treatment with MAC decreases IC_50_ of GEM almost 2.5-fold (Fig. 5b). Similar results were observed using the crystal violet viability assay. The application of GEM led to a reduction in cell viability, a effect that was even more pronounced when used in conjunction with MA (Fig. 5c). As expected, GEM prompted an increase in cl-PARP levels, a response that was enhanced with the administration of MAC. In order to observe how known markers of ET-1 activation behave, levels of ZEB1, β-catenin and NF-kB were measured, and a slight induction of both proteins was observed in the presence of GEM, which is reversed when blocking ET-1 receptors with MAC (Fig. 5d). Altogether, our results suggest that MAC may be a plausible therapeutic option in a synergistic sensitization of GEM effect in GBC cells (Fig. 6).

## Discussion

Our analysis revealed a significant and intriguing relationship between the expression of Endothelin-1 Receptor A (ET_A_R) and the median overall survival of GBC patients. Specifically, GBC patients with samples that displayed positive ET_A_R expression exhibited shorter survival durations. This finding strongly suggests that ET_A_R expression may serve as a valuable prognostic marker, indicating a poorer prognosis for those individuals with GBC. This observation aligns with previous research that has implicated ET-1 and its receptors in the progression and prognosis of various cancers [45, 52]. Conversely, in our study, we did not observe a statistically significant relationship between the expression of Endothelin-1 Receptor B (ET_B_R) and overall survival among GBC patients. This highlights, as in other types of cancer, that it is receptor A that would be more involved in the aggressiveness of cancers, without completely ruling out a possible role of receptor B [41]. This reaffirms the use of MAC to counteract the effect of both receptors or a compensatory effect when one of the two predominates.

Epithelial-to-mesenchymal transition (EMT) is a process which is characterized by loss of apical-basal polarity [53, 54] and cell–cell junctions [55, 56], synthesis and release of ECM-degrading metalloproteinases [57–59], increased migration and acquisition of invasive capacity [60–62], colonization of local and distant sites [63, 64], and enhanced chemoresistance [65–68]. Therefore, hindering and/or reverting EMT has been established as an approach to impair GBC invasion and metastasis, which would reduce the number of inoperable neoplasms. EMT is triggered in response to signals that cells receive from microenvironment, including Endothelin-1 (ET-1) [22, 34, 36]. In fact, it has been demonstrated that ET-1 promotes tumor migration [69], invasion [70], metastasis [71], stemness [72, 73], and chemoresistance [74].

Here, for the first time, it has been demonstrated the role of ET-1 in both downregulation of EMT-regulators, as well as migration and invasion of GBC cells. In fact, ET-1 extracellular levels were higher in metastatic cells (NOZ) in comparison to primary tumor cells (CAVE-1). Similar extracellular ET-1 levels have been observed in human tumor cell lines with epithelial-like morphology [75, 76]. High Endothelin-Converting Enzyme-1 (ECE-1) levels and low Neprilisin (NEP) levels might explain the aberrant ET-1 levels in NOZ cells. Thus, pharmacological modulation of ET-1 axis might impair GBC progression. However, we did not observe a positive correlation between ETRs protein and transcript levels. This disparity in mRNA and protein levels may be attributed to post-translational modifications, including phosphorylation, ubiquitination, glycosylation, and palmitoylation. These modifications hold the capacity to regulate the spatiotemporal dynamics of ETRs, consequently impacting their signaling and the resulting gene regulatory network during tumorigenic processes. Furthermore, it is noteworthy that various post-translational modifications can interact with each other, yielding both positive and negative effects.

ET-1 signals through its two G protein-coupled receptors (GPCRs): ET_A_R [77] and ET_B_R [78]. Many GPCR conformations lead to a variety of highly specialized downstream signaling cascades [79–83]. As a transducer downstream to ET_A_R, β-arrestin-1 translocates to the nucleus and interacts with β-catenin to promote target genes transcription (e.g.,* EDN1, AXIN2, MMP9* and *CCND1*) [84, 85]. Transcriptional activation of *EDN1* by β-catenin has been also observed in colon [86], prostate [87], and ovarian cancer [31] and creates a self-amplifying positive-feedback loop that forms an ET-1 autocrine circuit [22].

Here we have hypothesized that dual ETRs blockade with macitentan (MAC) may modulate ET-1 signaling downstream pathways in GBC cells. Blocking ETRs would induce changes in transcript levels of ET-1 signaling target genes, which would vary depending on the GBC cell line. In fact, in 1TKB cells*, VEGF* and *BIRC5* transcripts were reduced in presence of MAC. Likewise, *VEGF* and *CCND1* were downregulated in 2TKB cells with the same treatment. Consequently, we evaluated whether MAC treatment in NOZ, 1TKB and 2 KB cell lines modulated also β-catenin and NF-κB signaling. Total β-catenin and NF-κB protein levels were increased with ET-1 in NOZ cells and this was blocked upon MAC treatment, proving that MAC impairs ET-1-induced β-catenin and NF-κB signaling in a GBC in vitro model. In 1TKB cells, dual ETRs blockade reduced β-catenin nuclear levels. On the contrary, β-catenin cytosolic levels were not altered. In 2TKB cells treated with MAC, β-catenin was accumulated in the cytoplasm, where it might be bound with E-cadherin, thus involved in maintaining an epithelial phenotype. Additionally, activation of NF-κB is induced by ET-1 in various cancer cell lines [88–92], which has been involved with cell migration [93]. Here we demonstrated that NF-κB protein levels did not change after treatment with MAC in 1TKB and 2TKB cells, suggesting that ETRs dual blockade may be related to impairing β-catenin co-transcriptional activity and promoting binding with E-cadherin in 1TKB and 2TKB cells, respectively. Future studies should aim to elucidate if blocking ET-1 signaling hinders NF-κB or β-arrestins recruitment in GBC, which initiates signaling cascades in colorectal [67] and ovarian cancer [31]. The results from the Cycloheximide assay demonstrate that ET-1 plays a pivotal role in enhancing the stability of β-catenin, potentially influencing downstream signaling pathways. This finding indicates that ET-1 may contribute to the accumulation and maintenance of active β-catenin within cells. Significantly, the stabilizing effect of ET-1 on β-catenin is effectively countered when its receptors are blocked with MAC. Furthermore, the ability of ET-1 to increase the reporter activity of β-catenin, reversed by MAC, underscores the potential therapeutic implications of modulating β-catenin's activity in response to ET-1 and MAC, particularly in diseases associated with aberrant β-catenin signaling [13].

In order to assess whether MAC has a functional effect on cell migration and invasion, we tested the blockade of ETR on 3D-migration in cells of different aggressive origin. We found a correlation between the induction of E-cadherin at the protein level and the downregulation of ZEB1 and Snail, but no correlation was observed between mRNA and protein levels of some EMT-markers, suggesting a regulation at the level of protein stability and regulation of its degradation. No mRNA and protein levels of Twist (data not shown) were detected. As expected, cell invasion was considerably higher in 1TKB cells of metastatic origin compared to 2TKB cells originating from a primary tumor. In this sense, MAC decreases 1TKB invasion but has no effect on 2TKB. This suggests that blockade of ETRs affects the invasion of a cell highly invasive but with no effect on either non-invasive or minimally invasive cells.

Finally, we demonstrate that the widely used drug gemcitabine (GEM) reduces the viability of GBC cancer cells which is potentiated by blocking ETRs with MAC, indicating that MAC would be chemosensitizing these cells. Our results are similar to those found in pancreatic cancer [74], where it was shown that ET_A_R blockade sensitizes cells to GEM, however, in our model we have yet to understand the effect of each receptor separately. As observed in our study, the administration of GEM led to the expected increase in cleaved PARP (cl-PARP) levels, suggesting activation of apoptosis [74]. Interestingly, this response was further enhanced with the co-administration suggesting that ETRs blockage may potentiate the cytotoxic effects of GEM, possibly by intensifying the apoptotic response, which is in line with our expectations and adds a promising dimension to the use of these agents in combination therapy. Remarkably, the induction of β-catenin and NF-kB with GEM was reversed with MAC. This finding raises intriguing questions about the interaction between GEM and the ET-1 pathway. The reversal of protein induction by MAC suggest that the ET-1 pathway is involved in the observed responses to GEM. It's possible that ET-1 signaling is activated in response to GEM, leading to the induction of β-catenin and NF-kB. Subsequently, blocking ET-1 receptors with MAC could mitigate this activation, suggesting a potential mechanism for the chemosensitization effect we observed earlier.

Our study's findings are in line with existing research on the role of ET-1 in cancer, indicating both similarities and differences. Similarities the aberrant activation of ET-1 signaling in cancer development, such as tumor initiation, metastatic colonization and chemoresistance in several neoplasms [35]. Other similarity is the correlation between ETRs expression and pathological outcomes, such as patient survival and metastasis in various cancer models [36, 45]. However, the distinct tumor microenvironment, unique signaling networks, clinical presentation, and genetic variations in GBC may contribute to differences in ET-1's impact within this specific cancer. These differences are probably shaped by the distinct biology of GBC and could offer valuable insights for customized therapeutic strategies. Despite being relatively underexplored, similarities have been noted with gastrointestinal cancers, suggesting the potential for applying similar approaches to these tumor types [15]. Further research is needed to unveil the specific mechanisms behind these differences and to develop targeted treatments for GBC. The high mortality rate of GBC is largely due to silent and rapid progression as well as its marked aggressiveness and resistance to treatment [94, 95]. Altogether, we demonstrated that blocking ET-1 signaling hampers migration, invasion and chemoresistance in GBC cells, suggesting ETRs as novel therapeutic targets in GBC possible prognostic marker, which should be further confirmed in patient samples.

## Conclusions

The ET-1 signaling pathway is functionally active in gallbladder cancer (GBC) cells, and its extracellular levels positively correlate with increased malignancy. The pharmacological blockade of ET receptors (ETRs) using macitentan results in distinct regulation of nuclear protein levels of NF-κB and/or β-catenin, leading to altered expression of target genes. Furthermore, the inhibition of ET-1 signaling leads to the downregulation of epithelial-to-mesenchymal transition (EMT) markers, resulting in a reduction of cell migration and invasion capabilities. Importantly, ET-1 signaling blockade also enhances the chemosensitivity of GBC cells to gemcitabine. These findings collectively suggest that targeting the ET-1 axis represents a promising and novel therapeutic strategy for GBC treatment.

### Supplementary Information

**Additional file 1: Fig S1.** MAC do not affect cell viability of GBC cells. **a** NOZ were seeded and treated with 1 μM or 5 μM MAC for 72 h in the presence or absence of 1 mM pyruvate. Viability was indirectly measured by violet crystal staining and quantified by absorbance at 570 nm and plotted as percentage. **b** Same as in a, using 2TKB cells. **Table S1.** Primer sequences.

## Data Availability

Not applicable.
