# Supplementary material for: Targeting the Endothelin-1 pathway to reduce invasion and chemoresistance in gallbladder cancer cells
Source: Cancer Cell Int. 2023 Dec 10;23:318. doi: 10.1186/s12935-023-03145-9 (PMC10710704; doi:10.1186/s12935-023-03145-9)
Supplement: Supplementary file 1 — Additional file 1: Fig S1. MAC do not affect cell viability of GBC cells. a NOZ were seeded and treated with 1 μM or 5 μM MAC for 72 h in the presence or absence of 1 mM pyruvate. Viability was indirectly measured by violet crystal staining and quantified by absorbance at 570 nm and plotted as percentage. b Same as in a, using 2TKB cells. Table S1. Primer sequences. [file 12935_2023_3145_MOESM1_ESM.docx]

**Additional file**

**
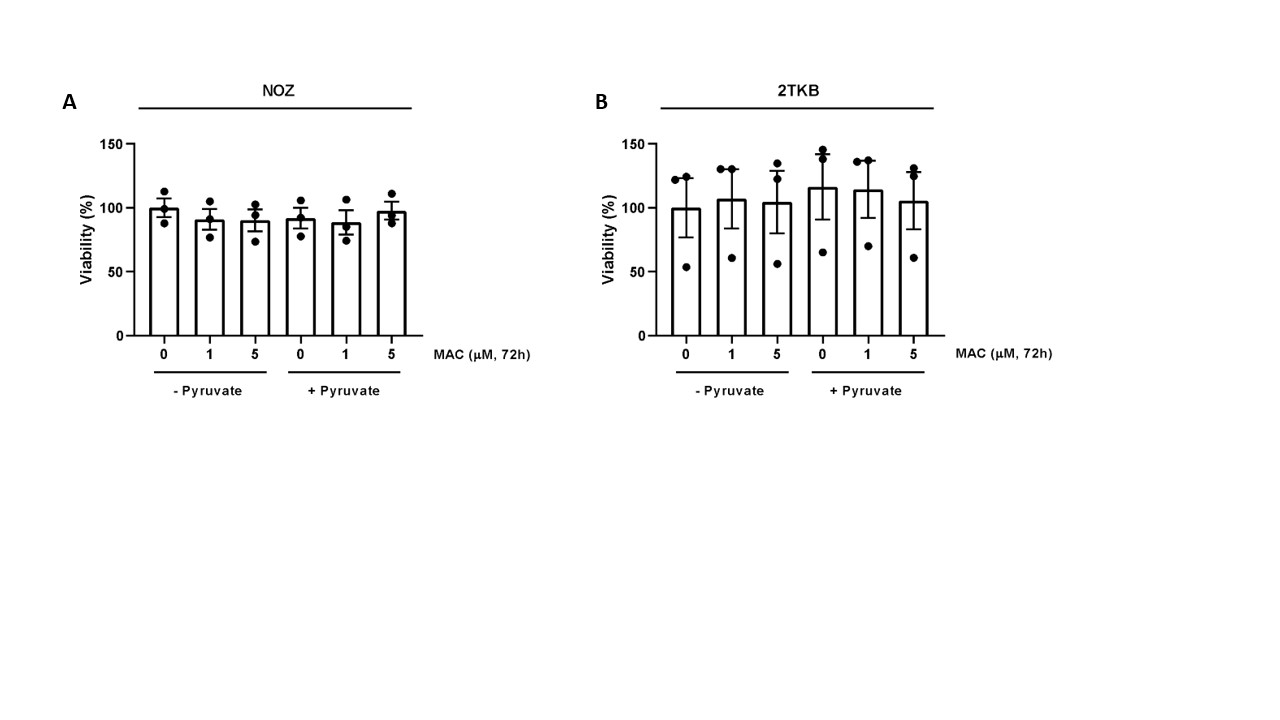
**

**Fig S1.** MAC do not affect cell viability of GBC cells. **a** NOZ were seeded and treated with 1 μM or 5 μM MAC for 72 h in the presence or absence of 1 mM pyruvate. Viability was indirectly measured by violet crystal staining and quantified by absorbance at 570 nm and plotted as percentage. **b** Same as in a, using 2TKB cells.

**Table S1.** Primer sequences

| **Gene** | **Forward primer sequence (5’-3’)** | **Reverse primer sequence (5’-3’)** |
| --- | --- | --- |
| *CDH1* | TCAAAGCCCAGAATCCCCAA | TCGGTTTTCTGTGCACACCT |
| *EDNRA* | TAAGAGGCAGCGTGAAAGCA | GCTCCAGCACAGGAAACAAT |
| *EDNRB* | ACTCCCAGGTAGGCATTTGC | TTCCTTCCCAGTGTTCAGCC |
| *SNAI1* | ACCTGTTTCCCGGGCAATTT | TAGTTCTGGGAGACACATCGGT |
| *TWIST1* | AGCCACTGAAAGGAAAGGCA | GGTTTTGCAGGCCAGTTTGA |
| *ZEB1* | ATGCAGCTGACTGTGAAGGT | TCATCCTCCCAGCAGTTCTT |
| *BIRC5* | TGCTAGAGCTGCCAGCTTTGTTCG | AGATTCAACAGGCACCTGCCAA |
| *CCND1* | GTGCCACAGATGTGAAGTTCATT | AAGTTGTTGGGGCTCCTCAG |
| *EDN1* | TGGGAAAAAGTGTATTTATCAGCA | TTTGACGCTGTTTCTCATGG |
| *MMP9* | ATTTCTGCCAGGACCGCTTCTACT | TGTCATAGGTCACGTAGCCCACTT |
| *SLUG* | CACTGCGATGCCCAGTCTAGAAAA | CTTGCCGCAGATCTTGCAAACA |
| *VEGF* | TTTCTGCTGTCTTGGGTGCA | CGCGAGTCTGTGTTTTTGCA |
| *ACTB* | AGAGCCTCGCCTTTGCC | TCACGCCCTGGTGCC |
